# Top-down evaluation of measurement uncertainty from validation data including sampling

**DOI:** 10.1007/s40199-026-00688-w

**Published:** 2026-10-29

**Authors:** Juliana Sasaki Ijiri, Felipe Rebello Lourenço

**Affiliations:** Universidade de São Paulo, São Paulo, Brazil

**Keywords:** Measurement uncertainty, Top-down approach, Sampling uncertainty, Method validation, Conformity assessment

## Abstract

**Background:**

Measurement uncertainty is essential for reliable pharmaceutical quality control and conformity assessment. However, conventional top-down approaches generally estimate uncertainty from validation data considering analytical variability, while uncertainty arising from sampling is frequently neglected.

**Objective:**

This study aimed to develop a practical top-down approach for evaluating measurement uncertainty from validation data that explicitly incorporates sampling uncertainty according to the principles of the Eurachem Validation of Measurement Procedures that Include Sampling (VaMPIS) guide.

**Methods:**

Precision was evaluated using a crossed–nested analysis of variance design involving target samples, independent replicate samples, intermediate precision conditions, and analytical replicates, enabling estimation of variance components associated with sampling, intermediate precision, and analytical repeatability. The trueness contribution to uncertainty was estimated from recovery studies. An MS-Excel workbook with automated routines was developed to support validation protocols and uncertainty calculations. The approach was applied to the assay of furosemide tablets by UV spectrophotometry and ibuprofen oral suspension by high-performance liquid chromatography.

**Results:**

Combined standard uncertainties of 3.4% and 3.1% were obtained for the furosemide and ibuprofen methods, respectively. The relative contributions to the uncertainty budget differed between the two procedures, while analytical repeatability contributed only marginally in both cases. The breakdown of precision uncertainty identified the principal sources of variability and supported targeted optimization strategies. Comparison with a target standard uncertainty of 3.2%, derived from the assay specification according to WHO recommendations, demonstrated the practical impact of including sampling in the uncertainty evaluation.

**Conclusion:**

The proposed approach provides a practical framework for integrating sampling uncertainty into pharmaceutical method validation, supporting Analytical Quality by Design principles and improving the reliability of conformity assessment and regulatory decision-making.

**Graphical abstract:**

**Figure:**
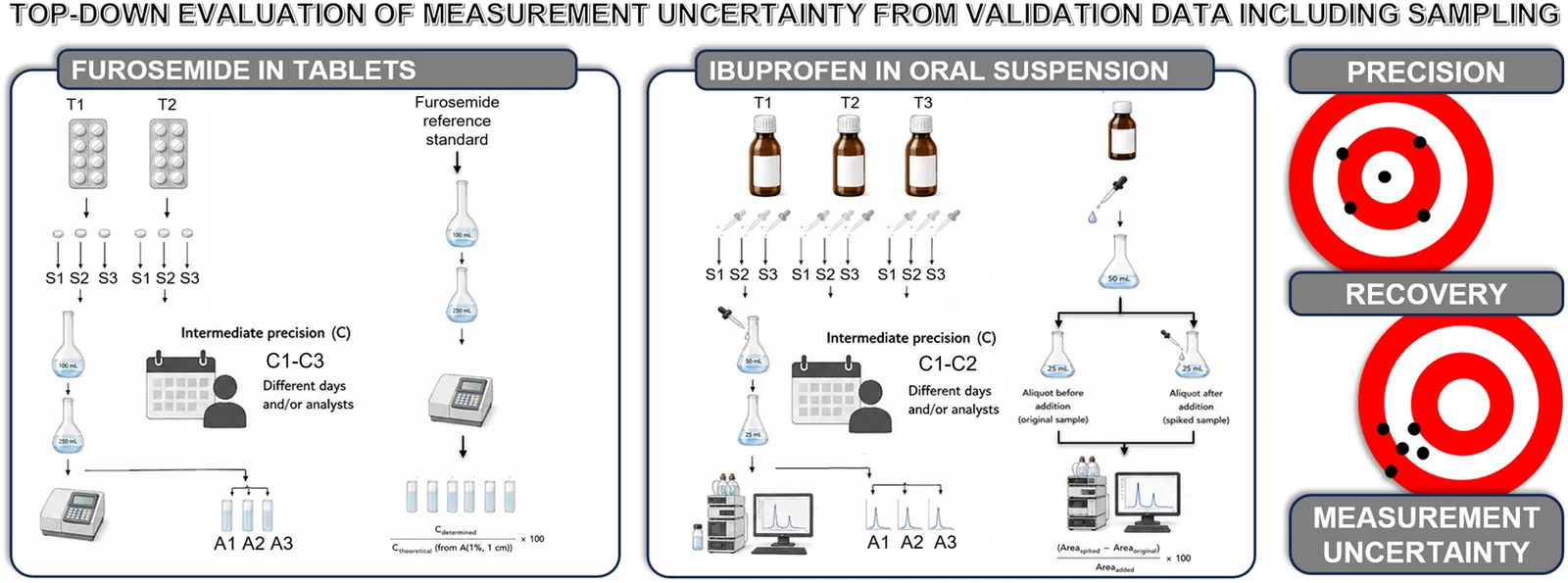

**Supplementary Information:**

The online version contains supplementary material available at https://doi.org/10.1007/s40199-026-00688-w.

## Introduction

Reliable analytical measurements are fundamental to assessing the quality, efficacy, and safety of medicines and pharmaceutical products throughout their lifecycle, from development to routine quality control and post-marketing surveillance [1–4]. Analytical results are routinely used to verify compliance with pharmacopeial specifications, support batch release, evaluate stability, establish pharmaceutical equivalence, and demonstrate regulatory compliance. Because the decisions based on these results may directly affect patient safety, product quality, and manufacturing costs, analytical results must be accurate. However, no measurement is exact, and every reported result is associated with measurement uncertainty, which represents the quantitative expression of the quality of the measurement result and the confidence that can be placed on it [5, 6].

Method validation is therefore an essential requirement for pharmaceutical analytical procedures. The recently revised ICH Q2(R2) guideline identifies precision and trueness (typically assessed through recovery studies) as fundamental validation characteristics [7], while ICH Q14 emphasizes the adoption of Analytical Quality by Design (AQbD) principles to develop robust analytical procedures supported by scientific understanding and risk management throughout the analytical lifecycle [8]. Within the AQbD framework, validation data are no longer viewed solely as evidence of method suitability but also as a valuable source of quantitative information for evaluating method performance and supporting science-based decision making [8–15].

Measurement uncertainty has become an integral component of modern analytical quality assurance because it summarizes the combined effects of random and systematic variability associated with a measurement procedure [5, 6]. Among the available approaches, the top-down strategy has gained widespread acceptance because it estimates measurement uncertainty directly from routine validation and quality control data, particularly precision and recovery studies, thereby reflecting the actual performance of the analytical procedure under laboratory conditions [16–19]. This approach is recommended by Eurachem/CITAC [20] and by the European OMCL Network [21] for pharmaceutical analyses. Nevertheless, conventional top-down approaches generally consider only analytical sources of variability, implicitly assuming that the test portion submitted to analysis is perfectly representative of the sampled material.

In many pharmaceutical applications, however, the sampling step may represent an important source of variability, particularly for heterogeneous dosage forms such as tablets, capsules, powders, and multiparticulate systems [22–24]. The Eurachem Guide Measurement Uncertainty Arising from Sampling [25] and the recently published Validation of Measurement Procedures that Include Sampling (VaMPIS) [26] emphasize that the measurement process begins with the selection of the primary sample and that validation should therefore encompass both sampling and analytical stages. Despite these recommendations, uncertainty arising from sampling is still rarely evaluated in pharmaceutical quality control laboratories, where validation protocols are usually restricted to analytical preparation and intermediate precision [7]. Consequently, the overall measurement uncertainty may be underestimated, potentially affecting the reliability of conformity decisions [25, 27].

The consideration of measurement uncertainty is particularly relevant during conformity assessment [5, 22]. Conformity decisions should be based not only on the measured value but also on its associated uncertainty through the application of predefined decision rules. The use of guard bands allows the establishment of acceptance limits that control the risks of false acceptance (consumer’s risk) and false rejection (producer’s risk), leading to more reliable regulatory and quality-control decisions [4, 5, 27–29]. Underestimated uncertainty values, especially when uncertainty arising from sampling is neglected, may result in an underestimation of these decision risks and consequently compromise confidence in batch acceptance decisions [22–24, 26, 30].

In this context, the present work proposes a practical approach for measurement uncertainty evaluation from validation data including the contribution arising from sampling. Precision studies were designed according to the principles of the Eurachem VaMPIS guide [26] using a hierarchical experimental structure capable of separating the uncertainty contributions associated with sampling, intermediate precision, analytical preparation, and instrumental repeatability, whereas recovery studies were used to estimate the uncertainty related to bias in the measurement result. Furthermore, an automated MS-Excel spreadsheet with embedded VBA routines was developed to generate standardized validation protocols for precision and recovery studies and to calculate the combined measurement uncertainty according to the top-down approach. The proposed approach provides a simple and practical tool for implementing uncertainty evaluation that is fully compatible with routine pharmaceutical method validation and supports risk-based conformity assessment. To the best of our knowledge, this is the first study that considers the principles of the Eurachem VaMPIS guide with the top-down estimation of measurement uncertainty from validation data, providing a practical approach for incorporating uncertainty arising from sampling into the measurement uncertainty associated with routine pharmaceutical analytical results.

## Materials and methods

In this work, two pharmaceutical products were analysed: (a) furosemide 40 mg tablets and (b) ibuprofen 100 mg/mL oral suspension. These two medicines were selected as case studies because of the distinct characteristics of their dosage forms (tablets and oral suspension), which may result in different contributions from sampling and analytical sources of uncertainty.

### Assay of furosemide in tablets by UV spectrophotometry

The assay was performed according to the Brazilian Pharmacopoeia monograph for furosemide tablets [31, 32], with minor adaptations. One tablet, corresponding to the labeled content of 40 mg of furosemide, was transferred to a 100 mL volumetric flask containing approximately 60 mL of 0.1 M sodium hydroxide. The mixture was shaken for 10 min, diluted to volume with the same solvent, homogenized, and filtered. A 5 mL aliquot of the filtrate was diluted to 250 mL with 0.1 M sodium hydroxide. A furosemide reference standard solution was prepared at the same concentration using 0.1 M sodium hydroxide. The absorbance of the sample and standard solutions was measured at 271 nm, using 0.1 M sodium hydroxide as blank. The furosemide content was calculated from the absorbance readings; alternatively, an absorptivity value of A(1%, 1 cm) = 580 at 271 nm may be used.

### Assay of ibuprofen in oral suspension by HPLC

The assay was performed according to the Brazilian Pharmacopoeia monograph for ibuprofen oral suspension [31, 32], with minor adaptations. Chromatographic analysis was carried out using an HPLC system equipped with a UV detector set at 220 nm and a 150 × 4.6 mm column packed with octylsilane-bonded silica (5 μm), maintained at room temperature. The mobile phase consisted of 0.01 M phosphoric acid and acetonitrile (63:37, v/v) at a flow rate of 2.0 mL/min. The diluent was acetonitrile–water (1:1, v/v). The oral suspension was thoroughly homogenized, and 12 drops, corresponding to approximately 60 mg of ibuprofen, were transferred to a 50 mL volumetric flask and diluted to volume with the diluent. The 12-drop aliquot was intentionally maintained as the test-portion sampling procedure to reflect the routine handling of the oral suspension. Therefore, variability associated with obtaining the aliquot was considered part of the sampling process rather than minimized through volumetric measurement. A 10 mL aliquot of this solution was transferred to a 25 mL volumetric flask and diluted to volume with acetonitrile. The standard solution was prepared similarly using ibuprofen reference standard at a final concentration of 0.48 mg/mL in the diluent. Following system suitability verification, 5 µL of the standard and sample solutions were injected separately, and the ibuprofen content was determined by external standardization based on the chromatographic peak areas of the sample and reference standard.

### Validation of measurement procedures including sampling

The validation study was designed according to the principles described in the Eurachem Validation of Measurement Procedures that Include Sampling (VaMPIS) guide [26], in which the measurement procedure is considered as an integrated process encompassing sampling, sample preparation, and instrumental analysis.

**Fig. 1:**
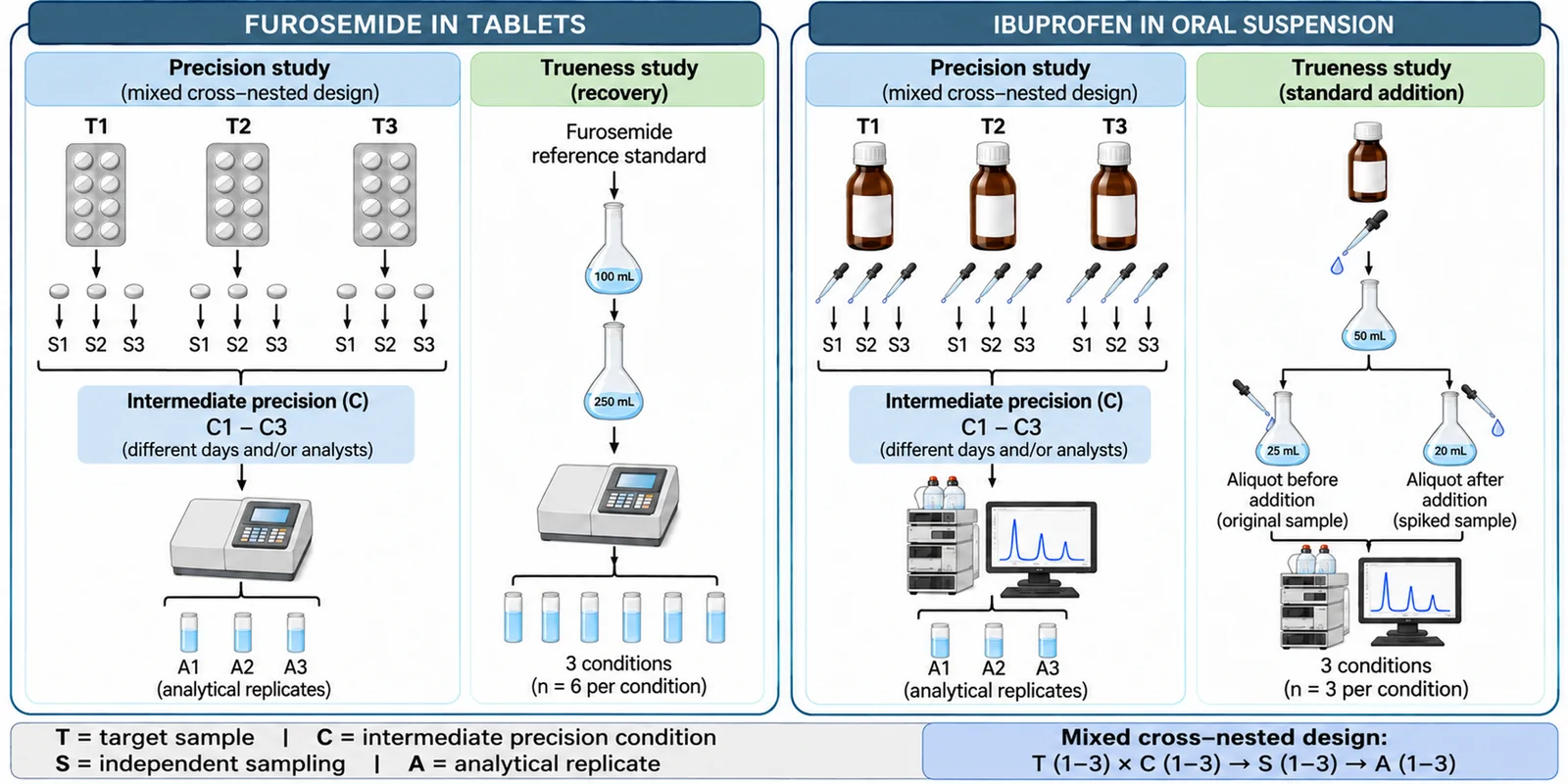
Experimental design used for validation and measurement uncertainty evaluation of the furosemide tablet and ibuprofen oral suspension methods. The precision study followed a mixed crossed–nested design comprising three target samples (T1–T3), three intermediate precision conditions (C1–C3), three independent samplings (S1–S3), and three analytical replicates (A1–A3). The trueness studies were performed using comparison with the pharmacopoeial specific absorptivity for furosemide and standard addition for ibuprofen

Accordingly, the precision study was designed to quantify the variance components associated with each stage of the measurement process, including target-to-target variance, sampling, intermediate precision, and analytical repeatability. For both the furosemide tablet and ibuprofen oral suspension methods, precision was evaluated using a mixed crossed-nested ANOVA design. Three independently selected target samples (T1-T3, blister packs for tablets and bottles for oral suspension) were analysed under three different conditions (C1-C3, different days/analysts). Within each target sample and condition, three independent samplings (S1-S3; individual tablets or 12-drop aliquots) were performed, and each preparation was analysed in triplicate (A1-A3). The experimental designs used for the precision and trueness studies are summarized in Fig. 1.

The number of observations required for reliable uncertainty estimation depends on the experimental design and the sources of variance to be estimated, and no single minimum design is applicable to all measurement procedures. ICH Q2(R2) recommends at least six determinations at 100% of the test concentration or nine determinations across the reportable range for repeatability, whereas the extent of intermediate precision studies should reflect relevant variations in routine use, such as days, analysts, and equipment. For measurement procedures including sampling, the experimental design should also provide independent replication at the sampling level. The Eurachem VaMPIS guide recommends at least eight sampling targets when the Duplicate Method is used; however, more structured designs may incorporate additional levels and replication. In the present study, the crossed–nested design comprised three target samples, three intermediate precision conditions, three independent samplings, and three analytical replicates, resulting in 81 observations per analytical procedure. This design provided replication at each level required for estimation of the corresponding variance components. Nevertheless, increasing the number of independent target samples and conditions would generally improve the reliability of variance-component and measurement uncertainty estimates.

Each blister pack containing ten furosemide tablets was considered a target sample. Three tablets were independently selected from each blister and analysed separately. Consequently, the sampling component represented the variance among individual tablets within the same blister and the dilution process, whereas the analytical component represented the variance associated with spectrophotometric measurement repeatability.

Likewise, each bottle containing ibuprofen oral suspension was considered a target sample. After homogenization, three independent test portions consisting of 12-drops each were collected. Accordingly, the sampling component represented the variance among independent 12-drop aliquots collected from the same bottle, including sample preparation (dilution), whereas the analytical component represented the variance associated with chromatographic measurement repeatability.

The mixed crossed–nested design enabled the estimation of variance components attributable to target samples, sampling, intermediate precision, and analytical steps using analysis of variance (ANOVA), thereby providing an estimate of the precision of the overall measurement procedure rather than that of the analytical step alone [23, 25, 30].

Trueness of the UV spectrophotometric method for furosemide tablets was assessed through recovery experiments performed under three intermediate precision conditions (different days and/or analysts), with six replicate determinations per condition. Bias was calculated by comparing the concentration determined from the reference standard solution with the theoretical concentration estimated from the specific absorptivity value (A(1%, 1 cm)) established in the Brazilian Pharmacopoeia. Trueness of the HPLC method for ibuprofen oral suspension was evaluated under three intermediate precision conditions (different days and/or analysts), each comprising three replicate determinations. Recovery was evaluated by standard addition. A known amount of ibuprofen reference standard was added to each sample aliquot, and the concentration measured before addition was subtracted from that measured after addition. Recovery was calculated as the percentage ratio between this experimentally determined increase in ibuprofen concentration and the known amount of ibuprofen added [7].

The target samples evaluated for each pharmaceutical product were obtained from the same production batch. Therefore, the experimental design was intended to characterize uncertainty components within a production batch rather than between-batch variability. The crossed–nested design, comprising three target samples, three intermediate precision conditions, three independent samplings, and three analytical replicates, provided replicated observations at each hierarchical level for estimation of the corresponding variance components. Nevertheless, the number of target samples was limited, and studies intended to characterize broader production variability should include a larger number of independently selected targets and, when relevant, multiple production batches.

### MS-Excel workbook for validation protocol and uncertainty calculations

An MS-Excel workbook with embedded VBA routines was developed to support the validation of analytical procedures, including the sampling steps. The macros were used to generate experimental protocols and to calculate uncertainty components associated with precision and recovery components.

The “ValidationProtocol_Precision” routine was used to automatically create the precision experimental layout in the worksheet “Precision Component”. The user defined the number of target samples, intermediate precision conditions, independent sampling and analyses replicates. Based on these inputs, the macro generated a crossed-nested design, assigning a unique identification code to each result according to the sequence target sample–condition–sampling–analysis. This structure allowed the recording of experimental values and ensured traceability of each measurement within the hierarchical validation design.

The “Uncertainty_Precision” routine was applied to calculate the uncertainty components associated with precision. The macro used the experimental results recorded in the “Precision Component” worksheet and performed a nested analysis of variance. The total variance was partitioned into components attributed to target samples, intermediate precision conditions, independent sampling and analyses. An auxiliary function calculated the sums of squares at each level, which were then used to obtain degrees of freedom, mean squares, variance components, and the corresponding standard uncertainty components. Negative variance estimates were set to zero. The combined precision uncertainty was calculated as the square root of the sum of the variance components.

The “ValidationProtocol_Recovery” routine was used to create the recovery experimental layout in the worksheet “Recovery Component”. The macro generated the combinations of matrix, concentration level, intermediate precision condition, and replicate, assigning a unique identification code to each recovery experiment. This structure was used to organize the recovery results obtained under different days and/or analysts and to support the subsequent estimation of recovery-related uncertainty components.

The “Uncertainty_Recovery” routine was used to evaluate recovery-related uncertainty. The macro calculated the mean recovery, the bias relative to the nominal recovery of 100%, and the standard deviation ($\:{s}_{Rec}$) of the individual recovery results. The standard uncertainty associated with the mean recovery was calculated as $\:{u}_{Rec}={s}_{Rec}/{n}_{R}$, where $\:{n}_{R}$ is the number of independent recovery determinations. The uncertainty associated with recovery bias was then estimated by combining the bias and the standard uncertainty of the mean recovery according to the law of propagation of uncertainty. An auxiliary function summarized the mean recovery values by matrix, concentration level, and intermediate precision condition. Although only one concentration level was evaluated for each analytical procedure in the present study, concentration level was included in the workbook structure to accommodate validation designs in which recovery is evaluated at multiple concentration levels. These summary statistics were intended to facilitate the interpretation of recovery performance and were not directly used in the uncertainty calculation. The estimated recovery bias uncertainty was subsequently used as the trueness-related component in the overall top-down measurement uncertainty evaluation.

The worksheet “Validation Protocol” was developed to integrate the results obtained during method validation and to calculate the combined standard uncertainty using the top-down approach. The worksheet automatically imports the uncertainty components calculated from the precision and recovery studies, allowing the user to define the validation model adopted for the uncertainty evaluation.

### Precision and trueness uncertainty components

The combined standard uncertainty ($\:{u}_{c}$) was calculated by combining the uncertainty associated with precision including sampling ($\:{u}_{p}$) and trueness ($\:{u}_{b}$) components, according to the Eq. (1) [20, 21]:

1 $$\:{u}_{c}=\sqrt{{{u}_{p}}^{2}+{{u}_{b}}^{2}}$$

Precision-related uncertainty component ($\:{u}_{p}$) was calculated according to Eq. (2) [20, 21] :

2 $$\:{u}_{p}=\sqrt{{{u}_{S}}^{2}+{{u}_{I}}^{2}+{{u}_{A}}^{2}}$$

where, $\:{u}_{S}$, $\:{u}_{I}$, and $\:{u}_{A}$, are the standard uncertainty components associated with sampling, intermediate precision, and analyses. Intermediate precision component.

Intermediate uncertainty component can be calculated as $\:{{u}_{I}}^{2}=\sqrt{{{u}_{C}}^{2}+{{u}_{TC}}^{2}}$, being $\:{{u}_{C}}^{2}=\left({MS}_{C}-{MS}_{TC}\right)/\left({n}_{T}\times\:{n}_{S}\times\:{n}_{A}\right)$ and $\:{{u}_{TC}}^{2}=\left({MS}_{TC}-{MS}_{S}\right)/\left({n}_{S}\times\:{n}_{A}\right)$, where $\:{MS}_{C}$, $\:{MS}_{TC}$, and $\:{MS}_{S}$ are the mean squares for conditions, interaction between target sample and condition, and sampling/sample preparation, respectively, and $\:{n}_{T}$, $\:{n}_{S}$, and $\:{n}_{A}$ are the numbers of target samples, sampling/sample preparations, and analyses [25, 26].

Sampling and analyses uncertainty components can be calculated as $\:{{u}_{S}}^{2}=\left({MS}_{S}-{MS}_{A}\right)/{n}_{A}$ and $\:{{u}_{A}}^{2}={MS}_{A}$, where $\:{MS}_{S}$ and $\:{MS}_{A}$ are the mean squares for sampling and for analyses, respectively, and $\:{n}_{A}$ is the number of analyses per sampling [25, 26] .

Trueness-related uncertainty component ($\:{u}_{b}$) was calculated according to Eq. (3) [20, 21] :

3 $$\:{u}_{b}=\sqrt{{b}^{2}+{{u}_{Rec}}^{2}}=\sqrt{{(1-\stackrel{-}{Rec})}^{2}+{\left(\frac{{s}_{Rec}}{{n}_{R}}\right)}^{2}}$$

where * b* is the observed bias ($\:b=1-\stackrel{-}{Rec}$), $\:\stackrel{-}{Rec}$ is the mean recovery, $\:{u}_{Rec}$ is the uncertainty of mean recovery ($\:{u}_{Rec}={s}_{Rec}/{n}_{R}$), $\:{s}_{Rec}$ is the standard deviation from recovery results, and $\:{n}_{R}$ is the number of recovery replicates.

The MS-Excel workbook containing the VBA routines developed for generating the validation protocols and performing the uncertainty calculations is provided as Supplementary Material to facilitate implementation and reproducibility of the proposed approach.

## Results and discussion

A summary of experimental results obtained during the precision and trueness studies are presented in Tables 1 and 2, respectively, for both furosemide tablets by UV spectrophotometry and ibuprofen oral suspension by HPLC analytical procedures.

**Table 1:** Precision results obtained for furosemide tablets by UV spectrophotometry and for ibuprofen in oral suspension by HPLC

		S1A1	S1A2	S1A3	S2A1	S2A2	S2A3	S3A1	S3A2	S3A3
Furosemide tablets	T1C1	97.8	97.8	98.0	100.8	101.2	100.6	102.8	104.9	103.4
	T2C2	97.1	96.9	97.5	98.5	98.5	98.5	96.0	96.7	96.4
	T2C3	91.9	91.7	91.9	98.0	98.3	98.3	93.6	98.2	98.7
	T3C1	102.0	101.3	101.1	99.9	101.2	100.1	99.0	99.0	99.3
	T3C2	98.3	99.4	98.1	95.8	97.2	96.2	99.4	101.3	101.7
	T3C3	97.9	99.6	98.7	94.2	94.2	94.2	100.8	101.5	101.5
		S1A1	S1A2	S1A3	S2A1	S2A2	S2A3	S3A1	S3A2	S3A3
Ibuprofen oral suspension	T1C1	105.7	105.7	105.7	108.2	108.0	107.9	107.8	107.9	108.2
	T1C2	109.5	109.4	109.4	110.4	110.5	110.4	108.9	108.9	108.9
	T2C1	103.6	103.6	103.7	106.2	106.2	106.3	105.9	105.7	105.9
	T2C2	107.4	107.0	107.1	110.2	110.1	110.1	110.0	109.8	110.0
	T3C1	109.0	109.0	109.1	109.4	109.5	109.2	109.4	109.0	109.0
	T3C2	108.7	108.4	108.3	110.7	110.9	110.9	109.9	110.0	110.0

Legend: T1, T2, and T3: target samples; C1, C2, and C3: intermediate precision conditions, S1, S2, and S3: sampling/sample preparation, and A1, A2, and A3: analyses. All values expressed as %

**Table 2:** Recovery results obtained for furosemide tablets by UV spectrophotometry and for ibuprofen in oral suspension by HPLC

		R1	R2	R3	R4	R5	R6
Furosemide tablets	M1L1C1	98.6	99.0	98.6	98.4	98.2	98.2
	M1L1C2	99.7	100.6	99.1	98.9	96.9	99.7
	M1L1C3	97.9	98.1	98.3	98.3	98.3	98.3
		C1R1	C1R2	C1R3	C2R1	C2R2	C2R3
Ibuprofen in oral suspension	M1L1	94.9	93.5	95.2	105.1	103.4	103.4
	M2L1	96.5	96.6	96.9	93.4	92.4	92.6
	M3L1	93.5	94.3	93.8	106.5	106.7	109.9

Legend: M1, M2, and M3: matrixes, L1: levels; C1, C2, and C3: intermediate precision conditions, and R1, R2, R3, R4, R5, and R6: instrumental replicates. All values expressed as %

The crossed–nested validation design provided replicated data at each experimental level for estimating the variance components used in the measurement uncertainty evaluation. These experimental results were subsequently processed using the proposed MS-Excel workbook to obtain the individual uncertainty components and the combined standard uncertainty presented in Table 3.

**Table 3:** Uncertainty components from precision and recovery studies calculated for furosemide tablets by UV spectrophotometry and ibuprofen in oral suspension by HPLC

Uncertainty component		Furosemide tablets by UV spectrophotometry	Ibuprofen in oral suspension by HPLC
Precision	$\:{u}_{I}$	1.8%	1.7%
	$\:{u}_{S}$	2.5%	1.2%
	$\:{u}_{A}$	0.9%	0.1%
	$\:{u}_{P}$	3.2%	2.1%
Recovery	* b*	1.2%	1.7%
	$\:\stackrel{-}{R}$	98.8%	98.3%
	$\:{u}_{R}$	0.2%	1.4%
	$\:{u}_{b}$	1.2%	2.2%
Combined	$\:{u}_{c}$	3.4%	3.1%

Legend: $\:{u}_{I}$ uncertainty from intermediate precision condition; $\:{u}_{S}$: uncertainty from sampling/sample preparation; $\:{u}_{A}$: uncertainty from analyses, $\:{u}_{P}$ uncertainty from precision, * b*: the bias (1$\:-\stackrel{-}{R}$), $\:\stackrel{-}{R}$: the mean recovery, $\:{u}_{R}$ uncertainty from mean recovery, $\:{u}_{b}$ uncertainty from bias, and $\:{u}_{c}$ combined standard uncertainty. All values expressed as %

Regarding the overall uncertainty budget, the precision component including sampling ($\:{u}_{p}$) represented the largest contribution to the combined standard uncertainty for both analytical procedures. The precision uncertainty, including sampling uncertainty, was 3.2% for the UV spectrophotometric determination of furosemide and 2.1% for the HPLC determination of ibuprofen, whereas the trueness component ($\:{u}_{b}$) was considerably smaller, corresponding to 1.2% and 1.7%, respectively. Consequently, the combined standard uncertainty was estimated as 3.4% for furosemide and 3.1% for ibuprofen. The relative contributions to the combined variance differed between the two analytical procedures. For furosemide, the precision-related variance was the dominant contribution (approximately 87%), whereas for ibuprofen, the variance associated with trueness was the dominant contribution (approximately 53%), with the precision-related variance accounting for approximately 47%. This observation is consistent with previous studies on top-down uncertainty evaluation, in which random effects obtained during method validation generally represent the dominant contribution to the uncertainty budget [16–19].

A detailed examination of the precision-related components provides insight into the origin of the observed variability and demonstrates the value of the proposed crossed-nested validation design. For the furosemide UV method, sampling represented the largest individual contribution to precision uncertainty (2.5%), accounting for approximately 62% of the total precision variance, whereas intermediate precision contributed 1.8% (31% contribution) and analytical repeatability only 0.9% (8% contribution). In contrast, the ibuprofen HPLC method was predominantly affected by intermediate precision (1.7%, corresponding to 67% contribution), while sampling uncertainty is 1.2% (33% contribution) and the analytical component was practically negligible (0.1%). The relatively large contribution of sampling to the uncertainty of the furosemide method indicates that the selection and preparation of individual tablets represent important sources of variability. The use of a composite sample prepared from multiple tablets could potentially reduce this contribution by averaging unit-to-unit variability. However, such an approach would modify the sampling procedure and should only be adopted when consistent with the intended measurand and routine analytical procedure.

These results indicate that the two analytical procedures were influenced by different sources of variability despite having similar validation designs. For the UV method, the analytical procedure itself was highly repeatable, and the remaining variability mainly reflected differences among individual tablets within the same blister. Likewise, intermediate precision was a dominant component for the ibuprofen procedure, indicating that routine day-to-day and/or analyst-to-analyst effects had a greater influence on the precision variance than analytical repeatability. This component may encompass variations associated with factors such as chromatographic conditions, solution preparation, instrument performance, and analyst-dependent operations, although their individual contributions were not separately estimated.

The uncertainty components estimated in this study were derived directly from validation data and therefore reflect the performance of the measurement procedures as implemented. The crossed–nested ANOVA allowed the precision contribution to be separated into sampling, intermediate precision, and analytical components, while recovery data were used to estimate the contribution associated with bias. Although the use of an internal standard could potentially reduce some sources of analytical variability in chromatographic procedures, particularly those related to sample preparation and injection, it is not required for estimating these uncertainty components using the proposed top-down approach. In the present study, the ibuprofen method was intentionally evaluated as routinely implemented using external standardization.

The separation of precision into sampling, intermediate precision, and analytical components also provides practical information for analytical quality improvement that cannot be obtained from conventional validation studies. Rather than reporting a single precision estimate, the proposed VaMPIS-based [26] approach identifies the stages of the measurement procedure responsible for the largest uncertainty contributions, thereby supporting targeted optimization strategies. For example, when sampling is an important uncertainty component, potential strategies may include increasing the representativeness of the test portion, such as using composite samples prepared from multiple units when consistent with the intended measurand, or improving the standardization of homogenization and aliquot collection for liquid formulations. Such modifications should be evaluated experimentally, since they alter the sampling procedure and may affect the measurand. Although the intermediate precision standard uncertainties were similar for the two procedures (1.8% for furosemide and 1.7% for ibuprofen), their relative contributions to the respective precision variances differed because of the different magnitudes of the other components. Thus, optimization priorities should be established considering the complete uncertainty budget of each measurement procedure rather than the magnitude of an individual component alone. The negligible contribution of analytical repeatability in both procedures further demonstrates that instrumental measurements were sufficiently precise and that additional replicate injections or absorbance measurements would provide little reduction in the overall uncertainty. Consequently, the proposed ANOVA-based breakdown of the precision variance into individual components not only provides a realistic estimate of the precision of the complete measurement procedure, as recommended by the Eurachem VaMPIS guide, but also establishes a rational basis for prioritizing method improvements within an Analytical Quality by Design (AQbD) framework [8].

The trueness study showed small bias values for both analytical procedures, corresponding to 1.2% for furosemide and 1.7% for ibuprofen, with mean recoveries of 98.8% and 98.3%, respectively. Nevertheless, the uncertainty associated with trueness ($\:{u}_{b}$) was approximately twice as high for ibuprofen (2.2%) as for furosemide (1.2%). This difference can largely be explained by the distinct recovery strategies adopted for the two analytical procedures. For furosemide, trueness was assessed by comparing the concentration determined from a reference standard solution with the theoretical concentration estimated from the specific absorptivity value established in the Brazilian Pharmacopoeia, resulting in relatively low variability among recovery determinations ($\:{u}_{Rec}=$0.2%). In contrast, recovery for ibuprofen was evaluated by the standard addition method using oral suspension samples that already contained the analyte. Consequently, the recovery calculation involved the difference between two experimentally determined concentrations (before and after addition of the reference standard), making the estimated recovery inherently more sensitive to random analytical variability. Moreover, any uncertainty associated with the quantification of the original ibuprofen concentration propagates directly into the recovery calculation, increasing the standard deviation of recovery results ($\:{u}_{Rec}=$1.4%) and, consequently, the trueness uncertainty [20, 21].

The practical relevance of the proposed uncertainty evaluation becomes evident when the results are interpreted in the context of conformity assessment. According to the recently revised WHO Good Practices for Pharmaceutical Quality Control Laboratories (WHO TRS 1052) [33], measurement uncertainty should be considered as an integral element of compliance testing because it enables laboratories to manage the risk of incorrect acceptance or rejection decisions [4, 33]. The guideline emphasizes that the pharmacopoeial decision rule remains a simple accept/reject decision based on the specification limits, provided that the analytical procedure operates within an acceptable level of uncertainty. Consequently, the estimated measurement uncertainty should not exceed the target uncertainty established for the analytical procedure; otherwise, the conformity decision should be regarded as inconclusive, requiring investigation of the causes responsible for the excessive uncertainty before reporting the analytical result.

For the assay methods evaluated in the present study, the specification limits were defined as 90–110% of the labelled amount, corresponding to a specification width of 20%. According to the concept of Normal Analytical Practice (NAP) described in WHO TRS 1052 [33], the target expanded uncertainty is approximately 32% o the specification width, corresponding to an expanded target uncertainty of 6.4%. onsidering a coverage factor of *k* = 2, the corresponding target standard uncertainty is therefore 3.2%, hich represents the maximum acceptable combined standard uncertainty for the analytical procedure to ensure conclusive pharmacopoeial compliance decisions.

It is worth noticing that the target uncertainty value is usually defined without considering uncertainty arising from sampling. In the hypothetical scenario where sampling uncertainty is neglected (or assumed to be negligible), the combined standard uncertainty would be 2.3% for the furosemide method and 2.8% for the ibuprofen method, both lower than the target uncertainty of approximately 3.2%.

These results highlight the practical importance of evaluating uncertainty arising from sampling. Although sampling is frequently neglected in conventional uncertainty evaluations, its contribution may substantially increase the overall measurement uncertainty and consequently affect the suitability of the complete measurement procedure, including sampling, for routine conformity assessment. A large sampling contribution does not necessarily indicate inadequate analytical performance, but may reflect heterogeneity among the sampled units or test portions that is captured by the analytical procedure.

When sampling uncertainty was incorporated into the uncertainty budget, the combined standard uncertainty increased to 3.4% for the furosemide measurement procedure, exceeding the target uncertainty of 3.2%. The dominant contribution originated from sampling ($\:{u}_{S}$=2.5%), indicating substantial tablet-to-tablet heterogeneity within the evaluated batch. Although modifications to the sampling strategy, such as increasing the representativeness of the test portion, may reduce its contribution to the uncertainty of the reported result, intrinsic product heterogeneity cannot be eliminated by optimization of the analytical procedure itself. In contrast, the combined standard uncertainty for the ibuprofen method was 3.1%, remaining within the target uncertainty and therefore satisfying the requirements of Normal Analytical Practice. For the furosemide method, the dominant contribution originated from sampling, indicating that increasing the number of independently sampled tablets would be an effective strategy to reduce the overall uncertainty. For example, analysing two independent samplings under repeatability conditions would reduce the combined standard uncertainty to approximately 2.9%, which is below the target uncertainty of 3.2%.

More importantly, the inclusion of sampling uncertainty may change the outcome of the uncertainty evaluation itself. An analytical procedure that appears suitable when only analytical uncertainty is considered may no longer meet the target uncertainty when uncertainty arising from sampling is incorporated into the overall measurement uncertainty Beyond identifying the principal sources of variability, the proposed approach explicitly incorporates the sampling stage into the uncertainty evaluation, in accordance with the principles of the Eurachem VaMPIS guide [26]. Consequently, the estimated uncertainty represents the complete measurement procedure rather than only the analytical steps. This distinction is particularly important in pharmaceutical quality control because the resulting uncertainty directly influences conformity assessment and the probabilities of false acceptance and false rejection of pharmaceutical products [5, 22, 24, 26, 34, 35].

## Conclusion

In conclusion, our findings demonstrate that measurement uncertainty should not be viewed exclusively as a statistical descriptor of analytical performance, but also as an objective quality indicator of the analytical procedure itself. Comparing the estimated uncertainty with the target uncertainty provides laboratories with a quantitative criterion for evaluating whether the analytical procedure can support reliable conformity assessment under routine conditions. This approach is fully aligned with the principles of Analytical Quality by Design (AQbD), since it identifies opportunities for continuous improvement of analytical procedures and establishes a direct relationship between validation performance, measurement uncertainty and confidence in regulatory decision-making.

## Supplementary Information

Below is the link to the electronic supplementary material.

Supplementary Material 1 (DOCX 13.4 KB)

## Data Availability

All data generated or analyzed during this study are included in this article and its supplementary information files.
