# Supplementary material for: Top-down evaluation of measurement uncertainty from validation data including sampling
Source: Daru. 2026 Oct 29;34(2):101. doi: 10.1007/s40199-026-00688-w (PMC13719957; doi:10.1007/s40199-026-00688-w)
Supplement: Supplementary file 1 [file 40199_2026_688_MOESM1_ESM.docx]

SUPPLEMENTARY MATERIAL LINK:

<https://sites.usp.br/labmeq/wp-content/uploads/sites/1641/2026/08/Top-Down_Uncertainty.zip>
